# Irritable bowel syndrome in adolescents in Lagos

**DOI:** 10.11604/pamj.2017.28.93.11512

**Published:** 2017-09-29

**Authors:** Oluwafunmilayo Funke Adeniyi, Olufunmilayo Adenike Lesi, Foluke Adenike Olatona, Christoper Imokhuede Esezobor, Joanah Moses Ikobah

**Affiliations:** 1 Department of Paediatrics, College of Medicine, University of Lagos,Lagos University Teaching Hospital, Idi-Araba, Lagos, Nigeria; 2 Department of Medicine, College of Medicine, University of Lagos, Lagos University Teaching Hospital, Idi-Araba, Lagos, Nigeria; 3 Department of Community Health, College of Medicine, University of Lagos, Idi-Araba, Lagos, Nigeria; 4 Department of Paediatrics, University of Calabar Teaching Hospital, Calabar, Cross River State, Nigeria

**Keywords:** Irritable bowel syndrome, adolescents, risk factors, subtypes

## Abstract

**Introduction:**

irritable bowel syndrome (IBS) is one of the functional gastrointestinal disorders (FGIDs) which has been well described in western populations especially as the commonest cause of recurrent abdominal pain The aim of this study was to document the prevalence of Irritable bowel syndrome (IBS) amongst children in western Nigeria and increase the aware ness of IBS amongst physicians who manage children with abdominal pain.

**Methods:**

This was a cross-sectional study conducted amongst children aged 10-18 years in 8 schools located in two local government areas of Lagos state. A multistage stratified random-sampling survey was conducted using the validated Rome III criteria to assess for IBS and associated risk factors. The subtypes of IBS and associated extra-intestinal symptoms were also documented.

**Results:**

The prevalence of IBS was 16.0% in the study participants and the prevalence decreased with increasing age (p=0.05). Sixty two (62.5%) of the students with recurrent abdominal pain had IBS. IBS was more prevalent in the females compared to the males (p=0.000). The significant risk factors for IBS identified were gender (p=0.000), socioeconomic status (p=0.001) and past history of gastroenteritis (p=0.011). The commonest subtype of IBS seen was the alternating subtype.

**Conclusion:**

IBS is prevalent in African children. Physicians who attend to children need to have a high index of suspicion for IBS in children who present with abdominal pain when there are no alarm symptoms. The need for further longitudinal studies in African children cannot be overemphasized.

## Introduction

Irritable bowel syndrome (IBS) is a disorder that has been categorized as one of the functional gastrointestinal disorders (FGIDs) which can occur in both adults and children [1,2]. According to the Rome III criteria, IBS is described as abdominal pain that improves with defecation and the onset of the pain is associated with change in stool frequency or consistency that cannot be explained by any biochemical or structural abnormality [3,4]. According to the Rome III criteria IBS can be sub classified depending on the predominant bowel habit into constipation IBS(C-IBS), diarrhea (D-IBS) and mixed or alternating IBS (A-IBS) where the patient has both constipation and diarrhea picture [4,5]. These subtypes presently have implications for effective therapeutic options. Studies done in the western world have observed that up to 50% of recurrent abdominal pain (RAP) is due to IBS [1-8]. However, regional variations in the prevalence of the condition has been reported [6]. There are few reports on FGIDs generally and especially on IBS in sub-Saharan Africa and the epidemiology of the illness is yet to be well elucidated in black African children. Some workers from southern Nigeria in a report on FGIDs in adolescents reported a prevalence of 5.6% for IBS amongst the FGIDs observed [9]. However, in this study and in most of the Asian studies, the subtypes of the condition were not described and some of the studies did not relate IBS in the context of RAP. The aim of this study is to document the prevalence of the condition and its subtypes in western Nigeria and increase the aware ness of IBS amongst physicians and care givers to include the condition in the differentials diagnosis of children who present with abdominal pain.

## Methods

**Study design and study location:** The study was carried out over a 6-month period (June-December) in 2015. It was a descriptive observational study involving children attending non-boarding secondary schools in Lagos metropolis. Lagos is the most populous city and the economic capital of Nigeria. Although the city derives its populace from many ethnic groups in Nigeria it is mostly inhabited by the Yorubas. The schools in the city are categorized into private funded and public funded schools depending on the ownership and the management or establishment bodies.

**Study procedure and ethical considerations:** The sample size was determined with the cochrane formula [10] for prevalence studies. The minimum sample size was calculated using 19.8% prevalence of IBS by Zhou et al [2] in Chinese urban adolescents, at 95% confidence interval, 5% degree of absolute precision with1.96 as standard normal deviation, and 20% attrition. The minimum sample size for each study site was 236. From a total of 20 local government areas (LGAs) in Lagos State, two LGAs were selected by ballot. A list of all the co-educational secondary schools in both LGAs was compiled and the schools categorized into private-funded and public-funded schools. Children attending private-funded schools belong, on the average, to families of higher socioeconomic class. Three and five schools were selected by ballot from the list of private and public-funded schools respectively. Each school has six levels (junior secondary school 1-3 and senior secondary school 1-3) and each level usually has between two and six arms. Depending on the school the number of children in each arm varies between 20 and 50 students with smaller numbers in private schools. The study population consisted of adolescents aged 10-18 years recruited from the (ie 5 public schools and 3 private schools) from the two local government areas. The study was approved by the Human Research Ethics Committee of the Lagos University Teaching Hospital. Prior to the commencement of the study further approval for the study was received from the State Education Board. With the assistance of the school principal and teachers each child was provided with a letter of informed consent to take home to their parent or guardians. Only those who returned a duly completed informed consent were enrolled in the study.

**Survey instrument:** The study was conducted using a questionnaire. The questionnaire contains section on the demographics of the child and the family, namely: age, parental education and occupation. Other information obtained were bowel habits, past history of gastroenteritis, oral regurgitation, dyspepsia, ingestion or preference for fried or spicy food, reported food allergy. Body weight, height and BMI were also documented. The second section of the questionnaire comprised the validated ROME III questionnaire for FGIDs for children above the age of 10 years [5,6] and determination of IBS was based on the specified criteria.

**Definition of terms:** The Rome III criteria [5,11] defines IBS as follows Abdominal discomfort or pain associated with 2 or more of the following (present at least 25% of the time): improved after defecation; onset of symptoms associated with a change in stool frequency; onset associated with a change in stool form alternating between diarrhea and constipation; no evidence of an inflammatory, anatomic, metabolic, or neoplastic process that explains the child's symptoms. The above criteria should be fulfilled at least once per week for at least 2 months before a diagnosis of irritable bowel syndrome is made.

**Alarm factors or red flag signs:** Presence of haematemesis, melena stools/blood in the stools, unexplained weight loss or anorexia, intractable vomiting, intestinal parasitemia prior to the commencement of the study, the Rome III questionnaire [5,6] was pretested with forty adolescents who attended 2 secondary schools (one private, one public) located in a LGA which was not used for the study. Twenty students were selected per school. All ambiguities were addressed and appropriate translations were done at this stage. After obtaining parental consent and assent from the students, a self-administered questionnaire was used to obtain information on demographic data, family structure and socio- economic status of the family and other required information. The questionnaires were filled with the guidance of the authors and four trained research assistants.

**Data collection :** A total of 2,500 questionnaires were administered, of these 2,000 questionnaires were appropriately filled giving a response rate of 80%. Subjects who met the Rome III criteria for IBS had physical examination and stools examinations done on them. Subjects identified with factors like unexplained weight loss, pallor, blood in the stools or intestinal parasites were excluded from the study and referred to LUTH for further review and management.

**Data analysis:** Data was analyzed with the SPSS version 21. Frequencies, proportions, means, and standard deviations [SD]) were calculated for all of the items of the questionnaire, Student t test was used to compare the continuous variables while categorical variables were compared using the chi square analysis. Logistic regression analysis was used to determine the factors associated with IBS and Unadjusted OR and 95% CI for IBS were also calculated. The level of significance was set at P < 0.05.

## Results

**Figure 1:**
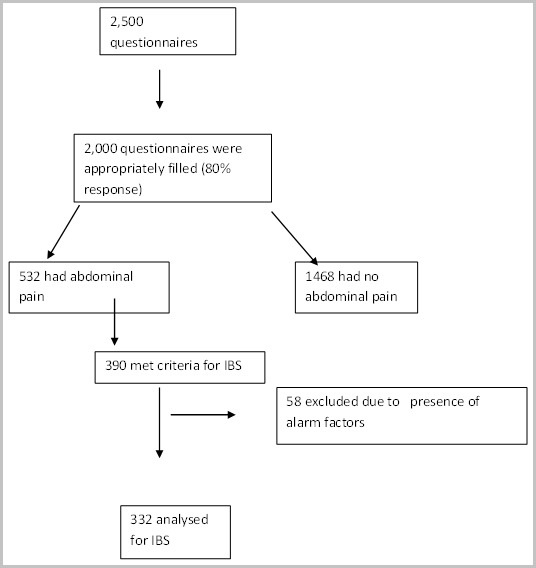
Profile of the study participants

2,000 students participated in the study, out of these 532 (26.6%) adolescents had abdominal pain but only 390 subjects met the criteria for IBS. However, 58 subjects were excluded due to the presence of alarm factors. Thus, only 332 of the subjects were analyzed for IBS in the study Figure 1.

**General characteristics of the study participants:** The ages of the participants ranged from 10 to 18 years with a mean (SD) of 13.23(2.04) years; Majority of the study participants were females (56.6%) and the male to female ratio was 1.0:1.3. Most of the participants were in the age group 13-15 years (45.8%) and belonged to the senior secondary school category (50.1%). The mean BMI (SD) of the study participants was 18.76(3.71). About a third (36.5%) of the students was from the low socioeconomic group. (see Table 1). Abdominal pain was reported by 532 (26.4%) subjects. 16.6% of the study participants had IBS.

**Table 1:** Clinical characteristics of the Study population

Parameter	Number (N)	Percentage (%)
**Age(yrs)**	N=2000	100
10-12	820	41.0
13-15	916	45.8
16-18	264	13.2
**Gender**		
Male	869	43.4
Female	1131	56.6
**Class**		
Junior Sec (JSS1-3)	1,031	50.1
Senior Sec (SS1-3)	999	49.9
**Socioeconomic Status**		
High	804	40.2
Middle	466	23.37
Low	730	36.5
**BMI percentile**		
<3^rd^ Centile	225	11.3
3^rd^-85^th^ centile	1,446	72.3
85th-97^th^centile	207	10.3
>97^th^ centile	122	6.1
Presence of Abdominal Pain	532	26.6
Presence of IBS	332	16.6

BMI=Body mass index, IBS- Irritable bowel syndrome, JSS-Junior secondary school, SSS-Senior secondary school

**Prevalence of IBS and characteristics of subjects with IBS:** in this study, 332 subjects were analyzed for IBS giving a prevalence of 16.6% in the participants Table 1. 532 subjects had abdominal pain, thus the prevalence of IBS within the study participants with abdominal pain was 62.5%. Table 2 shows the characteristics of the study population in relation to the presence or absence of IBS. The highest prevalence of the condition was observed in the age group 10-12 years (19.4%) and the lowest prevalence was seen in the children 16-18 years (13.7%) thus the prevalence decreased with increasing age in the participants. (p=0.018). IBS was more prevalent in the females (19.90%) compared to the males (12.3%). (p=0.0000) A significant proportion of the students from the low socioeconomic status (13.8%) had IBS compared to those from the middle class ((12.2%).There was a statistically significant difference in the socioeconomic status (p=0.000) between the subjects with and without IBS.

**Table 2:** Characteristics of the subjects with IBS and related factors

Parameter	Total	IBS(Yes)	IBS(No)	P value
**Age**		N (%)	N (%)	0.018
10-12	821	159(19.4)	662(80.6)	
13-15	916	137(15.0)	779(85.0))	
16-18	263	36(13.7)	227(86.3)	
**Gender**				<0.000
Male	868	107(12.3)	761(87.7)	
Female	1132	225(19.9)	907(80.1)	
**Socioeconomic status**				<0.000
High	804	175(21.8)	629(78.2)	
Middle	466	154(12.7)	407(87.3)	
Low	730	98(13.4)	632(86.6)	
**Preference for fried foods**				<0.000
Yes	332	59(17.8)	273(82.2)	
No	1668	274(16.4)	1394(83.6)	
**Past history of gastroenteritis**				0.000
Yes	513	227(44.2)	286(55.8)	
No	1487	105(7.1)	1,382(92.9)	
**Reported food allergy**				0.0001
Yes	816	156(19.1)	660(80.9)	
No	1184	151(12.8)	1,033(87.2)	
**Past history of dyspepsia**				0.0000
Yes	638	31(4.9)	607(95.1)	
No	1362	183(13.4)	1,179(86.6)	
**Drinking**				0.2026
Yes	86	2(2.3)	84(97.7)	
No	1914	105(5.5)	1,809(94.5)	
**Smoking**				0.1449
Yes	5	1(20)	4(80)	

IBS-Irritable bowel syndrome, p<0.05 is considered significant

**Risk factors and IBS:** The risk factors for IBS evaluated in the study participants are shown in tables Table 2 and Table 3. Univariate analysis showed that reported food allergy (p=0.025), past history of gastroenteritis (p=<0.0005) and dyspepsia (p=0.00001) were significantly related to the occurrence of IBS. However, multivariate analysis Table 3 showed that gender (p=0.00001), socioeconomic status (p=0.001), and past history of gastroenteritis (p=0.0005) were the only factors which could significantly predict the occurrence of IBS in the study population.

**Table 3:** rRsk factors for IBS: multiple logistic regression

Parameter	Regression coefficient	Standard Error	Wald	P value	Odd’s ratio	CI 95%CI
Age	0.026	0.038	0.457	0.499	1.026	0.952-1.106
Socioeconomic status	0.432	0.132	10.685	0.001	1.533	1.185-1.995
Gender	0.541	0.130	17.343	0.000	1.717	1.331-2.214
Past history of gastroenteritis	-0.349	0.137	6.464	0.011	0.705	0.539-0.923
Past history of dyspepsia	-0.162	0.127	1.904	0.168	0.839	0.654-1.076

**CI**: Confidence Interval, **IBS**: Irritable bowel syndrome, p value<0.05 is significant

**IBS Subtypes:** The IBS subtypes is shown in Table 4. The most prevalent subtype of IBS was the alternating IBS seen in 178(53.6%) of the subjects and the least prevalent was the diarrhea subtype seen in 63(19.0%) of the participants. The Alternating IBS was also significantly more prevalent in the females compared to the males. (p=0.00001).

**Table 4:** IBS subtype and gender

IBS subtype	Male	Female	Total	P value
	N (%)	N (%)	N (%)	
No IBS	761	907	1668	
IBS-A	49(27.5)	129(72.5)	178(53.6)	0.00001
IBS-C	36(39.6)	55(60.4)	91(27.4)	0.25552
IBS-D	21(33.3)	42(66.7)	63(19.0)	0.05369

IBS –A= Alternating irritable bowel syndrome, IBS-C= Constipation predominant irritable bowel syndrome and IBS-D= Diarrhea predominant irritable bowel syndrome

**Symptoms associated with IBS:** The extra intestinal symptoms associated with IBS observed in this study are highlighted in Table 5. Of all the extra intestinal symptoms observed, only difficulty with sleeping was significantly associated with IBS in the subjects (p=0.0000).

**Table 5:** IBS subtypes and extraintestinal symptoms

	IBS	Subtypes			
Extra intestinal symptom	ConstipationN (%)	DiarrheaN (%)	AlternatingN (%)	Total	Pvalue
	91(31.9)	63(17.9)	178(53.6)	332	
Difficulty with sleeping	61(26.4)	40(17.3)	130(56.2)	231	0.00001
Headaches	39(24.8)	27(17.2)	91(58.0)	157	0.112687
Back pain and pain in the arms and legs	40(23.8)	30(17.9)	98(58.3)	168	0.903523
Dizziness/fainting	48(26.8)	32(17.9)	99(55.3)	179	0.290892
School Absenteeism	42(25.1)	36(21.6)	89(53.3)	167	0.16143

IBS- Irritable bowel syndrome, p<0.05 is significant

## Discussion

In our study, 62.5% of the study participants with abdominal pain had IBS. This finding emphasizes the need for physicians to have a high index of suspicion for IBS in children who present with functional abdominal pain even in the sub-Saharan African subcontinent. The prevalence of IBS obtained in this study on urban Nigerian adolescents was 16.6% with the use of the Rome III criteria. This prevalence is higher than that obtained by Udoh et al (5.6%) [9] in south eastern part of Nigeria. In the latter study, a smaller cohort of adolescents from both rural and urban communities was observed and this may possibly explain the lower prevalence obtained. The prevalence in our study was also higher than some reports from Chinese and Italian children where a prevalence of 13.2% and 10.81%were obtained respectively [2, 12,13]. Most studies on IBS done in Asian countries report a prevalence ranging from 11%-22% in most of the school surveys [2,3,12,13]. These variations in the prevalence of IBS may be due to differences in selection criteria, racial, genetic, diet and possibly other peculiarities of the study population. Some workers have observed variations with the Rome criteria used [14,15]. Other reports have also documented a higher prevalence of IBS in studies conducted in hospitals compared to community studies [16,17]. This present study showed that the prevalence of IBS appear to decrease with increasing age (p=0.018). Zhu et al in a study on Chinese children aged 8-13 years [18] and Okeke et al [15] who documented IBS in a university student population in Nigeria have also observed a similar trend. The influence of gender on IBS has been reported in several studies and most authors report a higher prevalence in the females than males [2-4, 12-16]. Our study corroborates this observation (p=0.000) especially in subjects with the alternating IBS. This finding has been attributed to possible hormonal changes and it has been noted that the symptoms of IBS appear to be worse with menstrual cycles [18]. Nevertheless; some authors have observed that the condition was more prevalent in males while others have failed to report any significant gender differences with the condition [16,19].

In this study, there was a significant association of IBS with socioeconomic status and past history of gastroenteritis. This association corroborates various reports by researchers in Western populations [4, 12, 14,16, 18]. It is possible that children from low socioeconomic status who reside in areas with poor hygienic conditions, have poor sanitary habits, and be exposed to recurrent infections especially gastroenteritis which may also alter the gut biota in these children and cause abnormalities of enteric nervous system of the gut [4,12,14] . Other reported risk factors for IBS include food allergies and preference for fried foods [4, 12,14]. These factors however showed no significant risk association in this present study. Current evidence suggests that psychological insults during childhood may be associated with FGIDS [4, 12, 14]. And this was also observed by Udoh et al [9] in Nigeria. In view of the paucity of data on FGIDs and IBS in particular there is a need for more research to determine the risk factors for IBS in the African children in order for any other peculiarities to be identified and documented. The IBS subtypes and the presence of extra intestinal symptoms have important implications in the clinical management of these patients. In our study, the commonest subtype was the alternating IBS, more so in the female than male subjects. C-IBS has been observed to be the commonest subtype in some Italian children and in other parts of Europe [20-22]. Some reports have also noted that these subtypes may change with time in some children [21]. The role of diet, lifestyle and possibly racial factors in the distribution of IBS subclasses require further validation and study. IBS is a condition which has significant impact on the quality of life and this is more so when extra intestinal symptoms are present [4]. Extra intestinal symptoms seen in majority of the participants included difficulty with sleeping and headaches. School abseentism was also an important factor seen in 53.3% of the students with alternating IBS.

**Strength/limitations of the study:** This study involved a large cohort of adolescents in a community setting however there were few limitations the study. The subjects had to remember symptoms within the last 2 months and thus there lies the possibility of recall bias. Additionally, longitudinal follow up would have been desirable to in order to document any changes in the subtypes with time but this was not possible because of logistic difficulties.

## Conclusion

In summary, we have shown that IBS exists and is prevalent in African adolescents and a significant proportion of adolescents with abdominal pain have IBS. Furthermore, the association of lower socioeconomic level and past history of gastroenteritis were significant in this cohort. IBS was predominantly of the alternating type and was generally associated with significant extra intestinal symptoms. Nevertheless, physicians need to have a high index of suspicion for IBS in children who present with recurrent abdominal pain especially when there are no alarm symptoms. The need for further longitudinal studies in African children cannot be overemphasized.

### What is known about this topic

- IBS is a common cause of RAP in the caucascian population;
- Three subtypes of the condition has been described namely the C-IBS, D-IBS and A-IBS;
- Effective therapy may be related to the subtypes and possibly extra intestinal manifestations.

### What this study adds

- IBS is a prevalent condition in Nigerian adolescents;
- Socioeconomic status and past history of gastroenteritis were significantly associated with the occurrence of the illness in this cohort;
- Extra intestinal manifestations is a prominent feature of the illness in Nigerian adolescents.

## Competing interests

The authors declare no competing interests.
